# A c-di-AMP riboswitch controlling *kdpFABC* operon transcription regulates the potassium transporter system in *Bacillus thuringiensis*

**DOI:** 10.1038/s42003-019-0414-6

**Published:** 2019-04-29

**Authors:** Xun Wang, Xia Cai, Hongdan Ma, Wen Yin, Li Zhu, Xinfeng Li, Heon M. Lim, Shan-Ho Chou, Jin He

**Affiliations:** 1 State Key Laboratory of Agricultural Microbiology, College of Life Science and Technology, Huazhong Agricultural University, Wuhan, Hubei 430070 PR China; 2 Department of Biological Sciences, College of Biological Sciences and Biotechnology, Chungnam National University, Daejeon, 305-764 Republic of Korea; 3 Institute of Biochemistry and Agricultural Biotechnology Center, National Chung Hsing University, Taichung, 40227 Taiwan

**Keywords:** Bacterial genes, Riboswitches

## Abstract

The intracellular K^+^ level in bacteria is strictly controlled by K^+^ uptake and efflux systems. Among these, KdpFABC is a high-affinity K^+^ transporter system that is generally activated by the KdpDE two-component system in response to K^+^ limitation stress. However, the regulatory mechanism remains obscure in bacteria lacking the *kdpDE* genes. Here we report that the transcription of a *kdpFABC* operon is distinctively regulated by a cyclic diadenylate monophosphate (c-di-AMP) riboswitch located at the 5′-untranslated region of *kdp* transcript, and binding of c-di-AMP to the riboswitch promotes its intrinsic termination that blocks the *kdpFABC* transcription. Further, the intracellular c-di-AMP concentration was found to decrease under the K^+^ limitation stress, leading to transcriptional read-through over the terminator to allow *kdpFABC* expression. This regulatory element is found predominantly in the *Bacillus cereus* group and correlate well with the K^+^ and c-di-AMP homeostasis that affects a variety of crucial cellular functions.

## Abstract (web summary)

Xun Wang et al. show a *ydaO* riboswitch-regulated *kdp* operon expression in *Bacillus thuringiensis* BMB171. This work experimentally validates a long-suspected connection between the *kdpFABC* operon and the *ydaO* riboswitch identified on the genome of BMB171.

## Introduction

Potassium ion (K^+^) is one of the most abundant cations in the cells of all living organisms. It is essential for biological functions such as the regulation of osmotic pressure, membrane potential, acid–base homeostasis, gene expression, and cytoplasmic enzymatic activity^1–6^. To better carry out these functions, bacterial cells need to accumulate a high intracellular concentration of K^+^ from the very low level of this cation in the extracellular environment^7,8^. In bacteria, five classes of K^+^ uptake transporters have been described to date: the Kdp system, a recently discovered KimA (K^+^ importer A) protein, the Ktr and Trk system, and the Kup system^2,9,10^. Expression of the Kdp system is induced only under K^+^ limitation stress condition (lower than 2 mM)^11,12^. The Kdp system typically comprises four proteins: an ATPase KdpB and its chaperon KdpC, and a K^+^ transport protein KdpA, as well as a small accessory membrane protein KdpF^13^. Although the *kdpFABC* operons and structures of K^+^ uptake transporters are diverse, the regulation mode of *kdpFABC* operon appears to be rather consistent, being activated chiefly through a KdpDE two-component system^14^. Like other two-component systems, KdpD is a membrane-embedded histidine kinase sensor that auto-phosphorylates and transfers the phosphoryl group to the response regulator KdpE to activate the *kdpFABC* transcription under K^+^ limitation stress condition^14^.

Recent studies have demonstrated that the second messenger cyclic diadenylate monophosphate (c-di-AMP) is a crucial regulator for controlling K^+^ homeostasis. It controls K^+^ transporter activity or expression chiefly by binding to a receptor protein such as KtrA, TrkA, KdpD, c-di-AMP binding protein CabP, or cation/proton antiporter CpaA^15–17^. Binding of c-di-AMP to KtrA, TrkA, and CabP typically inactivates their transporter activities^15,18^. Similarly, interaction between c-di-AMP and KdpD UPS (universal stress protein) domain also suppresses the transcriptional activation of the *kdp* operon, thus reducing the amount of *kdp* transcript^19^. On the other hand, CpaA serves as an ion antiporter and binds with c-di-AMP to accelerate its K^+^ export activity^15,20^. Besides proteins, RNA can also form a stable three-dimensional (3D) structure such as the c-di-AMP riboswitch (previously called the *ydaO* riboswitch), which is a *cis*-acting RNA motif located at the 5′-untranslated region (5′-UTR) of a transcript for binding with c-di-AMP to regulate K^+^ transport^21^. A riboswitch often comprises two parts, an aptamer domain and an expression platform. As a genetic switch, binding of a specific ligand to the aptamer domain typically induces conformational changes in the expression platform, leading to different protein yield through transcriptional termination, activation/inhibition of translation initiation, or self-cleavage mechanism^21–25^. For example, a recent report showed that c-di-AMP was able to regulate the expression of a K^+^ transporter KimA through binding to a c-di-AMP riboswitch located at the 5′-UTR^10^. Besides, numerous putative c-di-AMP riboswitches located at the 5′-UTR of some K^+^ transporter transcripts (*ktr*, *trk*, *kdp*, and *kup*) have also been predicted in diverse strains such as phyla of *Firmicutes*, *Actinobacteria*, *Cyanobacteria*, *Proteobacteria*, *Verrucomicrobia*, and *Fusobacteria*^21,26^. Thus, c-di-AMP riboswitch seems to be an essential element in regulating K^+^ transporter expression in bacteria.

**Fig. 1:**
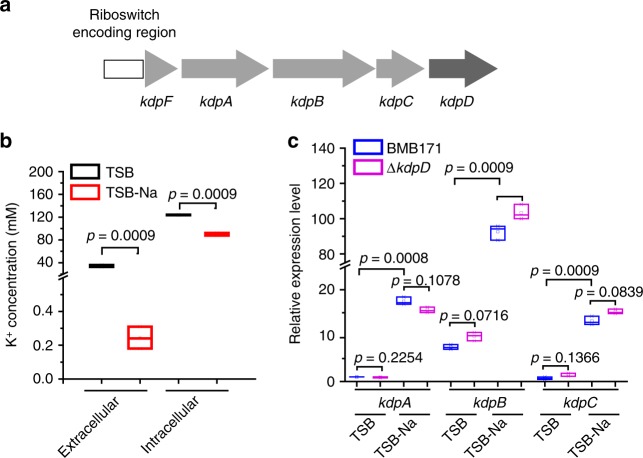
The *kdp* operon is induced under K^+^ limitation stress in BMB171**. a** A schematic presentation of *kdp* operon and *kdpD* gene. The cyclic diadenylate monophosphate (c-di-AMP) riboswitch encoding region is shown as a white rectangle, and the *kdpFABC* genes are depicted by thick light-gray arrows, while the *kdpD* by a thick dark-gray arrow. **b** Extracellular and intracellular K^+^ concentrations of BMB171 grown in TSB and TSB-Na. Data are expressed as box-and-whisker plots, where the central lines denote medians, edges represent upper and lower quartiles, and whiskers show minimum and maximum values. Data were subjected to one-way analysis of variance (ANOVA) using the Bonferroni test, *n* = 3; *p* values are shown above each panel. **c** Relative expression levels of *kdpA*, *kdpB*, and *kdpC* genes from BMB171 and Δ*kdpD* grown in TSB and TSB-Na. Data are expressed as box-and-whisker plots, where the central lines denote medians, edges represent upper and lower quartiles, and whiskers show the minimum and maximum values. Data were subjected to one-way analysis of variance (ANOVA) using the Bonferroni test, *n* = 3; *p* values are shown above each panel. Data underlying the plots in **b**, **c** are available in Supplementary Data 5

*Bacillus thuringiensis* is a well-known proteinaceous insecticidal crystal protein-producing strain^27^ that has been applied extensively in pest control. However, the mechanism of K^+^ transport in *B. thuringiensis* remains mostly unknown. *Bacillus thuringiensis* BMB171 is an acrystalliferous derivative strain of wild-type YBT-1463^28,29^, with a *kdp* operon comprising four protein-encoding genes of *kdpF* (*BMB171_RS03875*), *kdpA* (*BMB171_RS03880*), *kdpB* (*BMB171_RS03885*), and *kdpC* (*BMB171_RS03890*) (Fig. 1a)^30^. Moreover, a truncated *kdpD* (*BMB171_RS03895*) gene is found downstream of the *kdp* operon (encoding only 365 amino acids) that exhibits just low similarity to the N-terminal domain of *Staphylococcus aureus* KdpD (9.1%) (Supplementary Fig. 1). Nonetheless, to our surprise, we could not find any homologous *kdpE* gene in the BMB171 genome. These data suggest that the two-component KdpDE in BMB171 is likely defective. Instead, we found a c-di-AMP riboswitch sequence upstream of *kdp* transcript (Fig. 1a), which has also been predicted by a previous bioinformatic analysis^21^, hinting that BMB171 may incorporate a novel K^+^ uptake regulatory system for the *kdp* operon expression.

In this study, we reported a detailed analysis of the expression of *kdp* operon in BMB171 and found it was regulated uniquely via a c-di-AMP riboswitch. This work experimentally validates a long-suspected connection between the *kdpFABC* operon and the c-di-AMP riboswitch identified in the genome of BMB171.

## Results

### The BMB171 *kdp* operon expression upon K^+^ limitation stress

Two types of growth media, namely TSB (the K^+^ excess medium) and TSB-Na (the K^+^ limitation medium), were used to culture the bacterial cells^31^. We first measured the extracellular and intracellular K^+^ concentrations of BMB171 grown in these two media. Intriguingly, although there was a 163.1-fold difference for the external K^+^ concentrations at the different media (34.1 mM K^+^ in TSB versus 0.2 mM K^+^ in TSB-Na), only a 1.4-fold difference was observed for the internal K^+^ concentrations (124.0 mM versus 86.6 mM) (Fig. 1b). We also examined the transcription of the *kdp* operon after knockout of KdpD by real-time quantitative PCR (RT-qPCR). The experimental results showed that the expression levels of *kdp*A, *kdp*B, and *kdp*C between BMB171 and the Δ*kdpD* mutant were similar in either the TSB or TSB-Na (Fig. 1c). This aspect also indicates that knocking out KdpD does not seem to affect the transcription of the *kdp* operon. We then tested the inducibility of *kdp* operon under K^+^ limitation stress by RT-qPCR in both BMB171 and Δ*kdpD* strains. The RT-qPCR results showed that the *kdp* transcript was induced about 14-folds under a K^+^ limitation stress condition (Fig. 1c). These data confirmed our assumption that the *kdp* operon is inducible under K^+^ limitation stress. Moreover, the inducibility of the *kdp* operon in the absence of KdpD implied an alternative regulatory mechanism other than the well-studied KdpDE two-component system.

### A c-di-AMP riboswitch locates in the 5′-UTR of *kdp* transcript

**Fig. 2:**
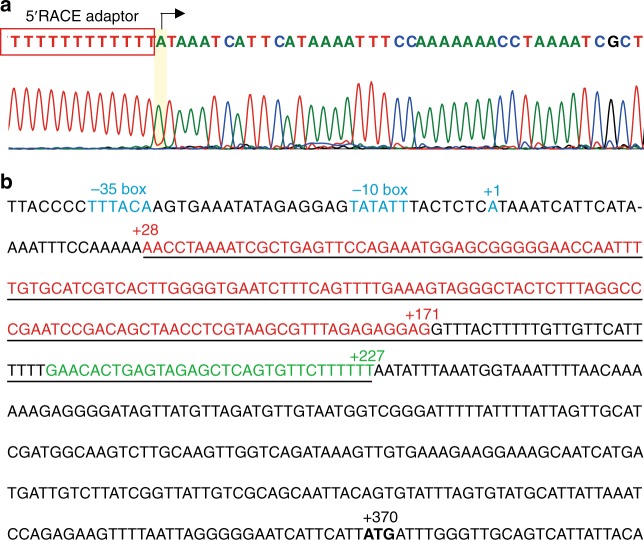
The sequence of the promoter and 5′-UTR encoding region of the *kdp* operon**. a** Mapping of the transcription start site (TSS) of *kdp* operon. The 5′-rapid amplification of cDNA ends (5′-RACE) adaptor sequence (boxed in red) along with the *kdp* transcript sequence after DNA sequencing were shown. The TSS is shaded in yellow. **b** The sequence of the *kdp* promoter (−43 to −1) and the 5′-UTR encoding region (+1 to +370). The −35 box and −10 box, as well as the +1 position, are marked in blue, and the cyclic diadenylate monophosphate (c-di-AMP) riboswitch encoding region is underlined (+28 to +227). Among them, the aptamer encoding region is marked in red (+28 to +171), while the intrinsic terminator in the expression platform encoding region (+172 to +227) is marked in bright green. The start codon ATG of *kdpF* is shown in bold

To explore how this *kdp* operon is regulated, we first identified the transcription start site (TSS) of the *kdp* operon by using the 5′-rapid amplification of complementary DNA (cDNA) ends (5′-RACE) method^32^. The first nucleotide base identified next to the oligo(dT)-anchor primer was an adenine residue (A) that was considered as the TSS of the *kdp* operon (Fig. 2a). Upstream of TSS, we identified a conserved −35 and −10 regions (both marked in blue). Importantly, the TSS residue A was found to be 370 bp upstream of the translation start codon of the *kdpF* gene, indicating a fairly long 5′-UTR encoding region (Fig. 2b). Blast search indicated that a c-di-AMP riboswitch encoding region (underlined) was present in the 5′-UTR encoding region (Fig. 2b), and the aptamer encoding sequence (Fig. 2b, 28–171 region, marked in red) of BMB171 is highly similar to that of *B. subtilis* 168 (67.6%) (Supplementary Fig. 2). The predicted secondary structures of the aptamer domain and expression platform (28–227 region, marked in bright green) of the c-di-AMP riboswitch are shown in Supplementary Fig. 2.

### The riboswitch regulates the BMB171 *kdp* operon expression

**Fig. 3:**
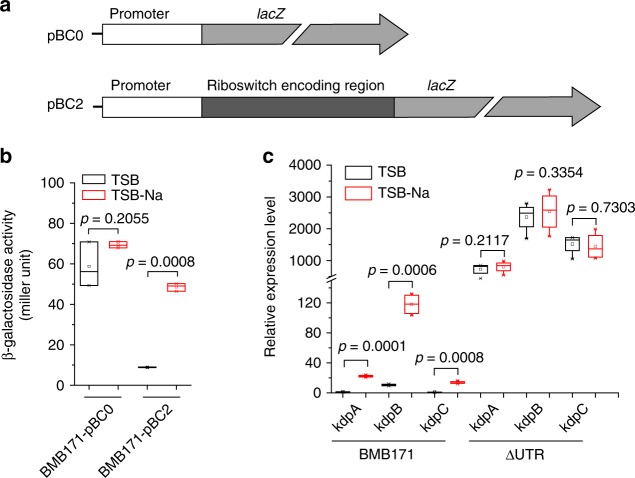
A cyclic diadenylate monophosphate (c-di-AMP) riboswitch transcriptionally regulates the *kdp* operon**. a** Scheme of plasmids pBC0 and pBC2 carrying a *lacZ* gene fused with the *kdp* operon promoter or the promoter with a c-di-AMP riboswitch, respectively**. b** The β-galactosidase activities for BMB171 carrying plasmids with (pBC2) and without the c-di-AMP riboswitch encoding region (pBC0) at different K^+^ concentrations. Data are expressed as box-and-whisker plots, where the central lines denote medians, edges represent upper and lower quartiles and whiskers show the minimum and maximum values. Data were subjected to one-way analysis of variance (ANOVA) using the Bonferroni test, *n* = 3; *p* values are shown above each panel. **c** Relative expression levels of *kdpA*, *kdpB*, and *kdpC* genes in the BMB171 and the ΔUTR strains measured by real-time quantitative PCR (RT-qPCR), respectively. Data are expressed as box-and-whisker plots, where the central lines denote medians, edges represent upper and lower quartiles, and whiskers show the minimum and maximum values. Data were subjected to one-way analysis of variance (ANOVA) using the Bonferroni test, *n* = 3; *p* values are shown above each panel. Data underlying the plots in **b**, **c** are available in Supplementary Data 5

Analysis of RNA secondary structure of the c-di-AMP riboswitch reveals a canonical intrinsic terminator in the expression platform comprising a conserved hairpin structure followed by a U-tract, which is located 143 bp upstream of the translation start site of *kdpF* transcript (Supplementary Fig. 2). Therefore, this c-di-AMP riboswitch probably controls the *kdp* operon expression through an intrinsic transcriptional termination mechanism. To verify its function, we first constructed two reporter assay plasmids of pBC0 and pBC2, in which either the *kdp* promoter alone (pBC0) or a *kdp* promoter along with a c-di-AMP riboswitch encoding region (pBC2) was fused to *lacZ*, respectively (Fig. 3a). While the β-galactosidase activities of BMB171-pBC0 in both K^+^-rich and -limiting conditions were similar, those of BMB171-pBC2 differed substantially in the two media (by 10-folds) (Fig. 3b). These results confirmed that the c-di-AMP riboswitch could sense the K^+^ limitation stress and activate the transcription of *kdp* operon. The β-galactosidase activities of the *kdp* deletion mutants of Δ*kdpD*-pBC0 and Δ*kdpD*-pBC2 grown in TSB and TSB-Na were also tested; however, no significant difference to those in the wild-type BMB171 could be observed (Supplementary Fig. 3). Thus, we confirmed that the *kdp* operon in BMB171 is regulated through a c-di-AMP riboswitch, but not by a KdpDE two-component system.

To further assess the role of the c-di-AMP riboswitch in regulation, we constructed a ΔUTR strain by only deleting the c-di-AMP riboswitch encoding region from the BMB171 genome and then quantitated the *kdp* transcripts by RT-qPCR. Upon its deletion, the overall expression level of *kdp* operon increased dramatically by 30-folds in average compared to that of BMB171 in TSB-Na, and the ΔUTR was no longer responsive to changing K^+^ concentrations (Fig. 3c). The data suggested that the presence of a c-di-AMP riboswitch reduced the *kdp* operon expression, and the switch was constitutively existent at an off state. However, under K^+^ limitation stress condition, *kdp* operon expression in the parent strain BMB171 could be induced to some extent.

### c-di-AMP represses the *kdp* operon expression

**Fig. 4:**
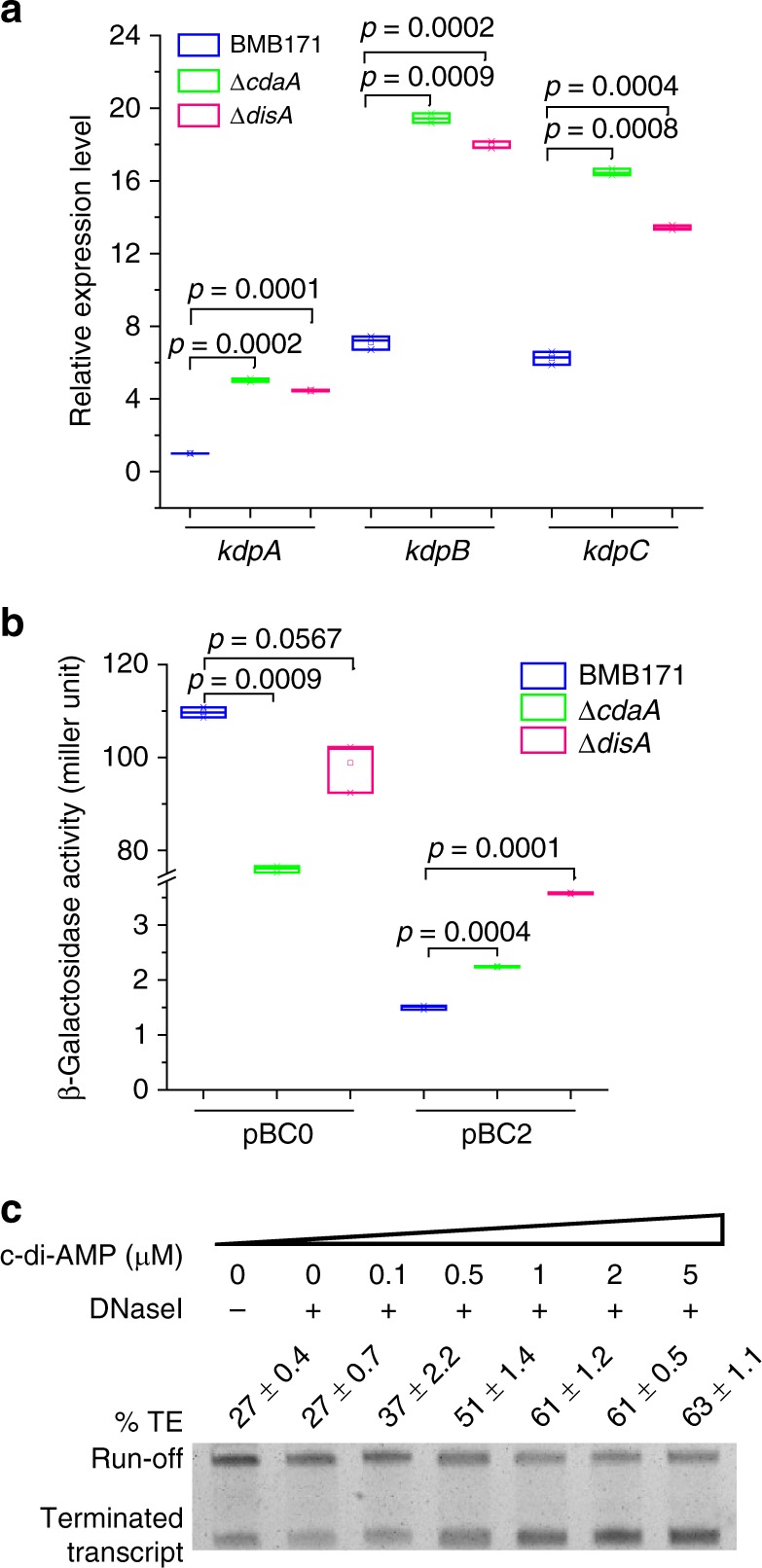
Influence of cyclic diadenylate monophosphate (c-di-AMP) concentration on the transcription of *kdp* operon**. a** Relative expression levels of *kdpA*, *kdpB*, and *kdpC* genes in the BMB171, Δ*cdaA*, and Δ*disA* strains grown in GYS measured by real-time quantitative PCR (RT-qPCR). Data are expressed as box-and-whisker plots, where the central lines denote medians, edges represent upper and lower quartiles, and whiskers show the minimum and maximum values. Data were subjected to one-way analysis of variance (ANOVA) using the Bonferroni test, *n* = 3; *p* values are shown above each panel. **b** β-Galactosidase activities for BMB171-pBC0, Δ*cdaA*-pBC0, Δ*disA*-pBC0, BMB171-pBC2, Δ*cdaA*-pBC2, and Δ*disA*-pBC2 strains were indicated in the figure. The results were given in Miller units. Data are expressed as box-and-whisker plots, where the central lines denote medians, edges represent upper and lower quartiles, and whiskers show the minimum and maximum values. Data were subjected to one-way analysis of variance (ANOVA) using the Bonferroni test, *n* = 3; *p* values are shown above each panel. Data underlying the plots in **a**, **b** are available in Supplementary Data 5. **c** In vitro transcription assay conducted in the presence of various c-di-AMP concentrations ranging from 0 to 5 µM. Termination efficiency (% TE) was calculated by the formula: ${\mathrm{Termination}}\,{\mathrm{efficiency}} = 100{\mathrm{\% }} \, \times \frac{{{\mathrm{Terminated}}\,{\mathrm{transcript}}}}{{{\mathrm{Terminated}}\,{\mathrm{transcript}} + {\mathrm{Run}} - {\mathrm{off}}}}.$

Next, we examined the c-di-AMP responsiveness of this riboswitch. BMB171 contains three diadenylate cyclases, including DisA (BMB171_RS00515), CdaA (BMB171_RS00930), and CdaS (BMB171_RS24565)^33^. In the GYS sporulation medium with K^+^ concentration at 7.9 mM, deletion of *cdaA* and *disA* gene reduced the c-di-AMP to approximately 80% and 74% of the original level, respectively^33^. After comparing the *kdp* operon expression levels between BMB171, Δ*cdaA*, and Δ*disA* in this medium by RT-qPCR, we found that the expression level of *kdpA* increased by approximately 4.5-folds, and those of *kdpB* and *kdpC* increased by 3-fold upon *cdaA* or *disA* deletion (Fig. 4a). We then measured the β-galactosidase activity of BMB171, Δ*cdaA*, and Δ*disA* harboring pBC0, and found that the c-di-AMP level only decreased slightly compared to that of BMB171-pBC0. However, the β-galactosidase activity increased by 1.4-folds in Δ*cdaA*-pBC2 and 1.8-folds in Δ*disA*-pBC2 compared to BMB171-pBC2 (Fig. 4b). These data proved that c-di-AMP works as a negative regulator for the *kdp* operon expression through a c-di-AMP riboswitch under the in vivo condition.

In vitro transcription was carried out by supplying c-di-AMP as a ligand and using a DNA fragment spanning from −88 to +363 (see Fig. 2a) as the template. Under increasing concentrations of c-di-AMP, the riboswitch-terminated transcripts were found to increase from 27% to 63%, with the run-off transcripts decreased accordingly (Fig. 4c and Supplementary Fig. 4). Therefore, c-di-AMP directly promotes the intrinsic termination of the riboswitch. This finding, together with the above in vivo results, confirmed that c-di-AMP represses *kdp* operon transcription through enhancing the transcriptional termination of a c-di-AMP riboswitch.

### Reduced c-di-AMP concentration enhances *kdp* operon expression

**Fig. 5:**
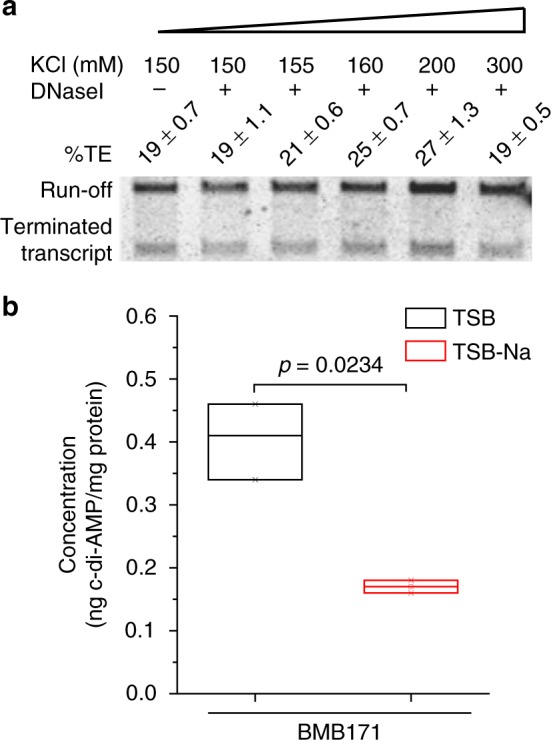
K^+^ indirectly regulates the expression of *kdp* operon**. a** In vitro transcription at increasing K^+^ concentrations ranging from 150 to 300 mM. **b** Intracellular cyclic diadenylate monophosphate (c-di-AMP) levels of BMB171 cells grown in TSB and TSB-Na. The c-di-AMP concentrations were determined by LC-MS/MS (liquid chromatography with tandem mass spectrometry). Data are expressed as box-and-whisker plots, where the central lines denote medians, edges represent upper and lower quartiles, and whiskers show the minimum and maximum values. Data were subjected to one-way analysis of variance (ANOVA) using the Bonferroni test, *n* = 3; *p* values are shown above each panel. Data underlying the plots in **b** are available in Supplementary Data 5

The above data showed that *kdp* operon transcription was activated under either K^+^ limitation stress or low c-di-AMP concentration. In other words, excess K^+^ or c-di-AMP represses *kdp* operon expression. Figure 4c demonstrates that c-di-AMP directly binds to the c-di-AMP riboswitch to regulate the intrinsic termination of the *kdp* operon transcription. However, how does K^+^ regulate the *kdp* operon transcription is currently unclear. There are two possibilities to explain this phenomenon. One is that K^+^ directly binds to the c-di-AMP riboswitch to regulate the intrinsic termination. The other possibility is that K^+^ indirectly regulates the *kdp* operon transcription through a c-di-AMP riboswitch mediated by c-di-AMP as has been described in *B. subtilis*^10^. To test these possibilities, in vitro transcription was performed with increasing K^+^ concentration ranging from 150 to 300 mM. However, the calculated termination efficiency did not seem to change much (Fig. 5a and Supplementary Fig. 5). Thus, it is unlikely that K^+^ directly binds to c-di-AMP riboswitch. The result was in good agreement with the previously reported crystal structure that K^+^ was absent in the atomic structure of c-di-AMP riboswitch^22,34^. We, therefore, considered another possibility that regulation of *kdp* operon expression by K^+^ is brought about indirectly by the different intracellular concentrations of c-di-AMP when K^+^ concentrations varied. We have thus quantified the intracellular c-di-AMP concentrations of BMB171 grown in TSB and TSB-Na by LC-MS/MS (liquid chromatography with tandem mass spectrometry). The results showed that the c-di-AMP level was down-regulated by 2.4-fold in TSB-Na relative to that in TSB (Fig. 5b and Supplementary Fig. 6). This result confirmed our prediction that c-di-AMP concentration was decreased under K^+^ limitation stress. Moreover, this result showed that a slightly (2.4-fold) reduced c-di-AMP level would lead to a greater change in gene expression (14-fold) (Fig. 1c). It supports well with the previous crystal structural observation that c-di-AMP bound to a c-di-AMP riboswitch at a 2:1 stoichiometry^22,34^. Thus, we could observe a larger change in gene expression for a smaller change in c-di-AMP. Taken together, we confirmed that the c-di-AMP riboswitch works as a transcriptional off riboswitch to regulate *kdp* operon expression. On the one hand, it entirely turns off *kdp* operon expression under high c-di-AMP concentration condition; on the other hand, a reduced c-di-AMP concentration under K^+^ limitation stress promotes read-through of the intrinsic terminator, thus increasing the *kdp* transcription.

### Distributions of regulatory elements of *kdp* operons

**Fig. 6:**
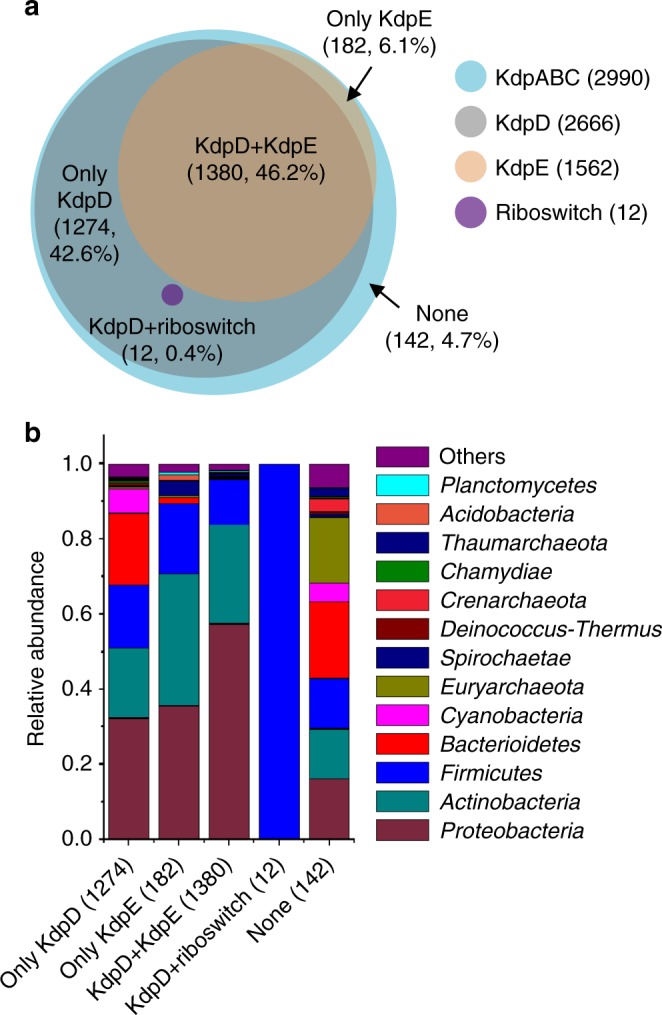
In silico analysis of Kdp system-related proteins and cyclic diadenylate monophosphate (c-di-AMP) riboswitch encoding region. **a** Three-way Venn diagram showing the numbers of KdpD (gray circle), KdpE (sandy brown circle) and c-di-AMP riboswitch (purple circle) that overlap in the bacterial and archaeal genomes containing KdpABC (sky blue circle). Numbers of bacteria contain both KdpD and KdpE (irregular saddle brown circle), only KdpD (cadet blue moon shape), only KdpE (burlywood moon shape), both KdpD and c-di-AMP riboswitch (purple circle), and none (sky blue irregular shape). **b** Comparison of each regulatory group in species from different phylogenic groups

To get an overview of the regulatory mechanism of *kdp* operon in the microbial world, we analyzed the distribution of KdpA, KdpB, KdpC, KdpD, and KdpE genes, as well as the c-di-AMP riboswitch encoding region in all sequenced bacterial genomes. This search revealed the presence of at least one homolog each for the KdpABC, KdpD, KdpE, and a c-di-AMP riboswitch-regulated *kdp* operon in 2990, 2666, 1562, and 12 different bacterial species, respectively (Fig. 6a, Supplementary Data 1). The species containing all the three proteins of KdpA, KdpB, and KdpC are considered to possess a potentially functional Kdp system, which appears to be present in most of the bacterial and archaeal phyla. However, in bacteria bearing the Kdp system (2990), only 46.2% (1380) of them contain both KdpD and KdpE (irregular turquoise circle) (Fig. 6a, Supplementary Data 1), while 42.6% (1274) contains only KdpD (green moon shape) (Fig. 6a, Supplementary Data 1) and 6.1% (182) only KdpE (cyan moon shape) (Fig. 6a, Supplementary Data 1). Thus, only about half of the species are regulated through the canonical KdpDE two-component system. Besides, 0.4% (12) (orange circle) of the species contain both KdpD and c-di-AMP riboswitch (Fig. 6a, Supplementary Data 1). Detailed analysis of these KdpD proteins revealed that they were in fact all truncated and contained the N-terminal domain only (Supplementary Data 2). It is worth noting that, none of the genome bearing an effective KdpDE system also contained a c-di-AMP riboswitch, suggesting that the c-di-AMP riboswitch and two-component KdpDE system are mutually exclusive for the *kdp* operon regulation. Moreover, 4.7% (142 out of 2990 genomes) (cornflowerblue irregular shape) of the species contain no KdpD, KdpE, nor c-di-AMP riboswitch (Fig. 6a, Supplementary Data 1), indicating the potential existence of other regulating elements for these species. Most of the species regulated by the two-component KdpDE are discovered in the phyla of *Proteobacteria*, *Actinobacteria*, and *Firmicutes* (Fig. 6b, Supplementary Data 1). However, the presence of *kdp* operon regulated through c-di-AMP riboswitch is mainly present in the *B. cereus* group, genus *Bacillus*, family *Bacillaceae*, order *Bacillales*, class *Bacilli*, phylum *Firmicutes*, and seems to be restricted to the order *Bacillales*, class *Bacilli*, and phylum *Firmicutes* (Supplementary Data 1 and Supplementary Data 3).

## Discussion

**Fig. 7:**
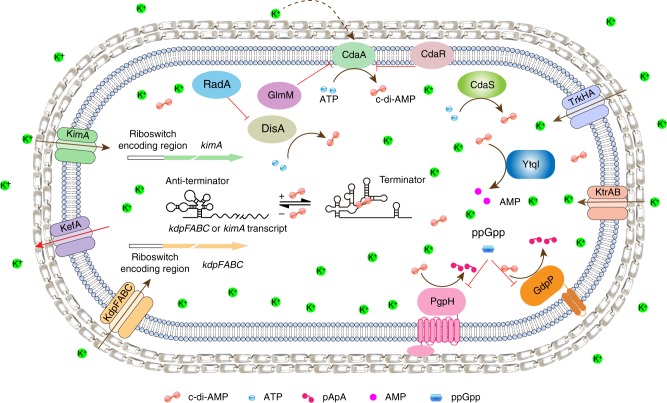
A proposed regulatory network between K^+^ transporters and cyclic diadenylate monophosphate (c-di-AMP) metabolic enzymes. Solid brown curved lines with arrowhead indicate enzymatic reactions, while dashed brown curved lines indicate indirect activation. Solid red lines without arrowhead indicate inhibition. Also, solid brown straight lines indicate K^+^ uptake and solid red straight lines indicate K^+^ efflux. c-di-AMP (red dumbbell) is synthesized by DisA, CdaA, or CdaS from two molecules of ATP (blue dot) and hydrolyzed by GdpP, PgpH, or YtpI to pApA (pink dumbbell) or AMP (pink dot). Factors spatially and temporally controlled the level of c-di-AMP by these enzymes may affect the cognate protein expression in transcription, translation, and so on. For examples, CdaR represses CdaA activity, GlmM represses CdaA, RadA inhibits DisA, (p)ppGpp (blue oval) inhibits GdpP and PgpH, K^+^ (green dot) down-regulates the protein level of CdaA. KimA (green complex), Trk (blue complex), Ktr (pink complex), and Kdp system (orange complex) are responsible for the K^+^ transport. KefA (purple complex) is responsible for K^+^ efflux. c-di-AMP binds to c-di-AMP riboswitch (black lines with clover structure) to promote transcriptional termination, thus down-regulating *kdp* operon and *kimA* expression

According to the genome annotation in the NCBI database, BMB171 possesses at least three kinds of K^+^ transporters. The K^+^ uptake transporter that actively transports K^+^ against a K^+^ gradient, including the Trk system (denoted by a green background in Supplementary Data 4), the Kdp system (indicated by a yellow background in Supplementary Data 4), and the KimA protein (indicated by a pink background in Supplementary Data 4). The K^+^ channel, in which K^+^ passes through the channel down the electrochemical gradient without the need for energy input, including Ktr system (indicated by a blue background in Supplementary Data 4) and some other K^+^ channels (denoted by an orange background in Supplementary Data 4). The K^+^ efflux system pumps out K^+^ (indicated by a gray background in Supplementary Data 4) (Fig. 7). We compared the K^+^ transporters between BMB171 and *B. subtilis* 168, the representatives of *B. cereus* and *B. subtilis* groups, respectively. *Baccilus subtilis* 168 seems to contain more K^+^ efflux systems (YugO, KhtUTS, and NhaK) and less K^+^ uptake systems (KtrAB, KtrCD, and KimA) (Supplementary Data 4)^10,35–37^, which indicates that the two main *Bacillus* groups in the genus *Bacillus* may have different habitations during evolution. In *B. subtilis*, the Kdp system is absent; instead, a KimA is responsible for the high-affinity transport of K^+^, while in BMB171, both high-affinity Kdp transport system and KimA are present. We searched the existence of *kimA* in eight species (168 strains) of *B. cereus* group (including *B. anthracis*, *B. cereus*, *B. cytotoxicus*, *B. mycoides*, *B. pseudomycoides*, *B. thuringiensis*, *B. toyonensis*, and *B. weihenstephanensis*), and found that *B. anthracis* (52 strains) and *B. cytotoxicus* (11 strains) do not contain any *kimA*, while *B. pseudomycoides* have two copies of *kimA*, and the other species only one (Supplementary Data 4). Interestingly, both *kimA* and *ktrAB* in the *B. subtilis* and *B. cereus* group are regulated through a c-di-AMP riboswitch^10^. Thus, the c-di-AMP riboswitches seem to play important roles in regulating K^+^ homeostasis.

We wondered whether there are any physiological advantages for adopting this special K^+^ regulatory mechanism. To date, four approaches controlling KdpD activity have been identified, including via K^+^ limitation stress, in which KdpD undergoes autophosphorylation;^38^ via dephosphorylation of the PTS system EIIA(Ntr) protein PtsN, in which the non-phosphorylated form interacts with KdpD;^39^ via UPS UspC, in which KdpD is activated and phosphorylated through interaction with its Usp domain;^40^ and via direct binding of c-di-AMP to KdpD to inhibit its activity^15^. From interacting with its partner proteins, the K^+^ uptake system may correlate with the nitrogen–carbon metabolism pathways and salt stress conditions. However, possible factors influencing the intracellular c-di-AMP level may be much more complicated than one expects, and we proposed that regulation through c-di-AMP or a c-di-AMP riboswitch enables the *kdp* operon to respond to a much larger arsenal of stimuli.

Among the c-di-AMP-producing strains, BMB171 is one of the strains containing the most abundant c-di-AMP metabolic enzymes. To date, there are two classes of enzymes responsible for controlling the intracellular level of c-di-AMP in strain BMB171, including the diadenylate cyclases DisA (BMB171_RS00515), CdaA (BMB171_RS00930), and CdaS (BMB171_RS24565), as well as the c-di-AMP-specific phosphodiesterases GdpP (BMB171_RS27370), PgpH (BMB171_RS21465), and YtqI (BMB171_RS22925)^33,41^. Multiple factors affecting the amounts and activities of these enzymes would thereby serve as the potential factors in altering the c-di-AMP concentrations. For example, when DNA damage occurs, damaged DNA may allosterically bind to DisA to inhibit the activity of DisA, leading to reduced c-di-AMP level^42^. Indeed, the DisA activity was also reported to be negatively affected by a DNA repair protein RadA^43^. Furthermore, another c-di-AMP synthetase CdaA was found to be regulated through interaction with CdaR or GlmM^44,45^, which is encoded in the same operon as CdaA. Since GlmM is essential for cell wall biosynthesis, c-di-AMP synthesis and peptidoglycan biosynthesis seem to be correlated through this kind of regulation. The third kind of c-di-AMP synthetase, CdaS, was also expressed explicitly in the sporulating cell via sigma factor *σ*^G^ or *σ*^H^^33,46^. Thus, synthesis of c-di-AMP also seems to be regulated by these growth-phase-dependent factors. As a c-di-AMP-specific phosphodiesterase, GdpP contains a PAS (Per-ARNT-Sim) domain, which recruits heme as a cofactor, enabling it to sense changes provoked by light, redox potential, and oxygen^47^. Meanwhile, (p)ppGpp could also act as a potent inhibitor of GdpP, and when cells were grown under a stringent condition, degradation of c-di-AMP was prevented^48^. Furthermore, (p)ppGpp also inhibited the hydrolysis activity of another c-di-AMP-specific phosphodiesterase, PgpH; thus, a strict response was essential for maintaining c-di-AMP homeostasis^49^ (Fig. 7). In summary, it is conceivable that through direct regulation by c-di-AMP, K^+^ uptake is linked to the central cellular process and is well correlated with specific cellular function.

Many types of researches have been carried out to explain how c-di-AMP affected the K^+^ homeostasis; however, the mechanisms by which K^+^ regulates c-di-AMP concentration remain mostly unknown. A recent study has reported that in strain *B. subtilis* 168, the amount of CdaA was decreased under K^+^ limitation stress, leading to a diminished c-di-AMP level^10^. Another study in *Streptococcus pneumoniae* reported that the deletion of a K^+^ transporter-encoding gene *cabP* reduces its intracellular c-di-AMP levels^50^. As discussed above, there are six c-di-AMP metabolic enzymes in BMB171, and there are also various potential kinds of proteins and small molecules to inhibit or activate these enzymatic activities. Intracellular K^+^ concentrations may also control the transcriptions, translations, and activities of those enzymes. Thus, K^+^ would directly or indirectly influence the synthesis and degradation of c-di-AMP. The regulatory network between K^+^ and c-di-AMP appears to be complicated, and there seems to have no simple answer on how K^+^ regulates the c-di-AMP homeostasis to date. Further study is required to answer this issue.

Based on the above descriptions, we propose a regulatory network between K^+^ transporters and c-di-AMP metabolic enzymes in BMB171. BMB171 possesses three diadenylate cyclases and three c-di-AMP-specific phosphodiesterases, which can respond to diverse signals to efficiently control the synthesis and degradation of c-di-AMP. For example, binding of RadA to DisA reduces its c-di-AMP synthesis capability, and binding of CdaR or GlmM to CdaA also inhibits its c-di-AMP synthesis activity. Further, the amount of CdaA is reduced under K^+^ limitation stress. On the other hand, (p)ppGpp binding inhibits the activities of GdpP and PgpH. The four primary K^+^ uptake transporters are drawn in Fig. 7. Among these transporters, the *kdp* operon and *kimA* are regulated through a c-di-AMP riboswitch. Thus, factors influencing c-di-AMP homeostasis can act indirectly to regulate the *kdp* operon expression through a c-di-AMP riboswitch to regulate the K^+^ homeostasis (Fig. 7). It has been reported that K^+^ concentration, as well as expression of Kdp system, were required to affect the production of toxins and virulence factors in *S. aureus*, *Salmonella typhimurium*, *Yersinia pestis*, *Acinetobacter baumannii*, *Streptococcus pneumoniae*, *Mycobacterium tuberculosis*, and so on^51–55^. Therefore, understanding the regulatory mechanism of K^+^ homeostasis in pathogens is essential for developing an effective and innovative method to prevent bacterial-infective and malignant diseases.

## Methods

### Plasmids, bacterial strains, and growth conditions

The strains and plasmids, as well as the primers used in this study, were listed in Supplementary Tables 1–3. *Escherichia coli* DH5α was used for cloning experiments. *Escherichia coli* strains were cultured at 37 °C in lysogeny broth (LB) medium (g L^−1^: tryptone, 10; yeast extract, 5; NaCl, 10). When necessary, appropriate antibiotics were added to the cultures at the following final concentrations: 50 μg mL^−1^ for kanamycin, 25 μg mL^−1^ for erythromycin, 300 μg mL^−1^ for spectinomycin, or 60 U for polymyxin. BMB171 and its derivative strains (except Δ*cdaA* and Δ*disA*) were cultured at 28 °C in the media of TSB (g L^−1^: glucose, 2.5; casein peptone, 17.0; soy peptone, 3.0; K_2_HPO_4_·3H_2_O, 3.3; NaCl, 5.0) and TSB-Na (g L^−1^: glucose, 2.5; casein peptone, 17.0; soy peptone, 3.0; Na_2_HPO_4_·12H_2_O, 5.1; NaCl, 5.0)^31^. For the RT-qPCR and β-galactosidase activity analysis of Δ*cdaA* and Δ*disA* mutant, cells were cultured at 28 °C in GYS (g L^−1^: glucose, 1; yeast extract, 2; K_2_HPO_4_·3H_2_O, 0.655; (NH_4_)_2_SO_4_, 2; MgSO_4_·7H_2_O, 0.041; MnSO_4_·H_2_O, 0.0378; CaCl_2_, 0.08).

### RNA extraction, cDNA synthesis, and RT-qPCR

The total sample volumes of 15 mL each from BMB171 and its derivative strains cultured in either the TSB, TSB-Na for 6 h or GYS for 11 h were centrifuged, with cell pellets ground in liquid nitrogen. Total RNA was isolated, and RT-qPCR was performed as described previously^33,56,57^. In those experiments, either the *gapdh* or 16S rRNA gene was used as an internal control.

### Identification of TSS

The 5′-RACE experiment was performed as described previously with modifications^32^. RNA was extracted from BMB171 cells grown in TSB-Na, followed by reverse transcription to cDNA. The 3′ end of cDNA was labeled by poly(dA) using terminal deoxynucleotidyl transferase (Takara, Japan). The cDNA was then PCR amplified using primers listed in Supplementary Table 1. PCR products were cloned to pMD19-T vector (Takara, Japan) and sequenced.

### Construction of the ΔUTR and Δ*kdpD* mutants

The mutants Δ*kdpD* and ΔUTR were constructed by the markerless gene deletion method as described earlier^33,58,59^. Deletion of these genes was confirmed by sequencing of the PCR fragments amplified using primers listed in Supplementary Table 1. Sequence alignment of PCR products amplified from the Δ*kdpD* and ΔUTR genomic DNA and the BMB171 genomic DNA were shown in Supplementary Fig. 7. Strains were listed in Supplementary Table 2.

### Construction of transcriptional fusion plasmids

The 100 bp *kdp* promoter, 269 bp promoter, and c-di-AMP riboswitch coding regions were amplified using primer pairs listed in Supplementary Table 1. The PCR products were digested with corresponding restriction enzymes and ligated into the plasmid pHT1K-*lacZ*, which was constructed in our laboratory previously^60,61^. The corresponding plasmids were named as pBC0 and pBC2, respectively (Supplementary Table 3). After confirmation by sequencing, the plasmids were extracted from DH5α and electroporated into the corresponding *B. thuringiensis* strains.

### Determination of β-galactosidase activity

BMB171-pBC0 and BMB171-pBC2 strains were grown at 28 °C in an orbital shaker at 200 r min^−1^ in 100 mL TSB or TSB-Na with 25 µg mL^−1^ erythromycin. Two milliliters of cultures were collected at 6 h (mid-log phase) and were assayed for β-galactosidase activity as described previously^60,61^. Δ*cdaA*-pBC0, Δ*cdaA*-pBC2 and Δ*disA*-pBC0, and Δ*disA*-pBC2 strains were grown at 28 °C in an orbital shaker at 200 r min^−1^ in 100 mL GYS with 25 µg mL^−1^ erythromycin. Two milliliters of cultures were collected at 11 h (mid-log phase) and were assayed for β-galactosidase activity.

### Determination of K^+^ concentrations

Bacterial cells were cultured in TSB and TSB-Na for 6 h at 28 °C. Fifty milliliters cultures were harvested, with the intracellular K^+^ concentrations determined using an atomic absorption spectrometer (Upper Bio-tech, Shanghai, China) as described previously^10,62^. The intracellular concentrations of K^+^ were calculated using the following equation:

$$\left[ K \right]_{\mathrm{i}} = \frac{{\left[ K \right]_{\mathrm{t}}}}{{\left( {W_{\mathrm{w}} - W_{\mathrm{d}}} \right) \div {\mathrm{\rho }}}},$$

in which [*K*]_i_ is the intracellular K^+^ concentration in mM, [*K*]_t_ is the total K^+^ in µmol, *W*_w_ is the wet weight of the pellet in, *W*_d_ is the dry weight of the pellet in g, and *ρ* the density (1 g mL^−1^).

To better determine the K^+^ concentrations in TSB and TSB-Na, as well as GYS, 100 µl medium was mixed with 5 mL of 65% HNO_3_ with the following steps performed as described above. The concentrations of K^+^ were calculated using an equation of the following type:

$$\left[ K \right]_{\mathrm{e}} = \frac{{\left[ K \right]_{\mathrm{t}}}}{V},$$

in which [*K*]_e_ is the extracellular K^+^ concentration in mM, [*K*]_t_ is the total K^+^ concentration in µmol, and *V* the volume of the medium in mL.

### Quantification of intracellular c-di-AMP concentration

c-di-AMP was detected and quantified using a liquid chromatography coupled with triple quadrupole tandem mass spectrometry LCMS-8040 (Shimadzu, Japan) by the same protocol as published previously^41^. The observed peak for ion 135.9*m*/*z* was integrated. The level of the nucleotide was determined by comparing the integrated peak area to a calibration curve generated using a c-di-AMP sample purchased from Merck & Co., Inc. (Merck, USA). Protein concentrations were determined by NanoDrop (Thermo Fisher Scientific, USA).

### In vitro transcription

The PCR fragment containing the *kdp* promoter and c-di-AMP riboswitch encoding region was served as a DNA template for in vitro transcription. The reaction was performed using *E. coli* RNA polymerase (NEB, USA) according to the manufacturer’s instructions. Briefly, DNA template (2 nM) was incubated at 37 °C for 5 min in reaction buffer (40 mM Tris-HCl, pH 7.5; 10 mM MgCl_2_; 150 mM KCl; 1 mM dithiothreitol [DTT] and 0.01%Triton X-100) containing 2 U RNA polymerase and 40 U RNase inhibitor (Promega, USA) in an 18 μl reaction. c-di-AMP was added to final concentrations of 0, 0.1, 0.5, 1, 2, and 5 μM. The reaction was initiated by the addition of 2 μl NTP mix (final concentration, 0.1 mM each NTP) to the mixture. After incubation at 37 °C for 1h, DNase I (1 U) was added, with the reaction continued for 20 min and finally terminated by the addition of formamide loading buffer (90% formamide, 10 mM EDTA, 0.01% xylene cyanol, and 0.01% bromophenol blue). Samples were heated at 95 °C for 3 min before loading onto the 6% acrylamide/8 M urea gel, which was stained with ethidium bromide and then visualized. When required for reaction with increasing K^+^ concentration, reaction buffer (40 mM Tris-HCl, pH 7.9; 6 mM MgCl_2_; 1 mM DTT and 2 mM spermidine) were used, with 1 M KCl added to get the final concentrations at 150, 155, 160, 200, and 300 mM.

### Bioinformatic analyses

To analyze the distribution of *kdp* genes, we searched each protein with keywords of KdpA, KdpB, KdpC, KdpD, and KdpE in NCBI, UniProt, and KEGG database. For the confirmation of KdpD candidate proteins, each of the 27 KdpD amino acid sequences was used as the query. BlastP searches were performed using default parameters with a cutoff identity value of 40% and a bit score of 500. The c-di-AMP riboswitch was searched against Rfam. Results were compared and synchronized.

### Reporting summary

Further information on research design is available in the Nature Research Reporting Summary linked to this article.

## Supplementary information

Reporting Summary

Description of Supplementary Data

Supplementary Information

Supplementary Data 1

Supplementary Data 2

Supplementary Data 3

Supplementary Data 4

Supplementary Data 5

## Data Availability

All data generated or analyzed during this study are included in this published article, its Supplementary Information, and Supplementary Data files. The source data underlying each plot are presented in Supplementary Data 5.
