# Publisher Correction: A c-di-AMP riboswitch controlling *kdpFABC* operon transcription regulates the potassium transporter system in *Bacillus thuringiensis*

**DOI:** 10.1038/s42003-019-0449-8

**Published:** 2019-05-09

**Authors:** Xun Wang, Xia Cai, Hongdan Ma, Wen Yin, Li Zhu, Xinfeng Li, Heon M. Lim, Shan-Ho Chou, Jin He

**Affiliations:** 1 State Key Laboratory of Agricultural Microbiology, College of Life Science and Technology, Huazhong Agricultural University, Wuhan, Hubei 430070 PR China; 2 Department of Biological Sciences, College of Biological Sciences and Biotechnology, Chungnam National University, Daejeon, 305-764 Republic of Korea; 3 Institute of Biochemistry and Agricultural Biotechnology Center, National Chung Hsing University, Taichung, 40227 Taiwan

**Keywords:** Bacterial genes, Riboswitches

Correction to: *Communications Biology* 10.1038/s42003-019-0414-6, published online 29 April 2019

In the original published version of the article, the country names for affiliations 1 and 3 were incorrect. The error has been corrected in the HTML and PDF versions of the article.

