# Supplementary material for: A c-di-AMP riboswitch controlling kdpFABC operon transcription regulates the potassium transporter system in Bacillus thuringiensis
Source: Commun Biol. 2019 Apr 29;2:151. doi: 10.1038/s42003-019-0414-6 (PMC6488665; doi:10.1038/s42003-019-0414-6)
Supplement: Supplementary file 2 — Description of Supplementary Data [file 42003_2019_414_MOESM2_ESM.docx]

**Description of Additional Supplementary Files**

**File Name**: Supplementary Data 1

**Description**: Lists of bacterial species possessing Kdp elements.

**File Name**: Supplementary Data 2

**Description**: Lists of KdpD sequences in 12 representative strains from 12 species.

**File Name**: Supplementary Data 3

**Description**: List of K^+^ transporters.

**File Name**: Supplementary Data 4

**Description**: Lists of strains from *B. cereus* group in which *kdp* operon is regulated through c-di-AMP.

**File Name**: Supplementary Data 5

**Description**: All source data underlying the graphs.
