# Supplementary material for: A c-di-AMP riboswitch controlling kdpFABC operon transcription regulates the potassium transporter system in Bacillus thuringiensis
Source: Commun Biol. 2019 Apr 29;2:151. doi: 10.1038/s42003-019-0414-6 (PMC6488665; doi:10.1038/s42003-019-0414-6)
Supplement: Supplementary file 3 — Supplementary Information [file 42003_2019_414_MOESM3_ESM.pdf]

Supplementary Figure 1

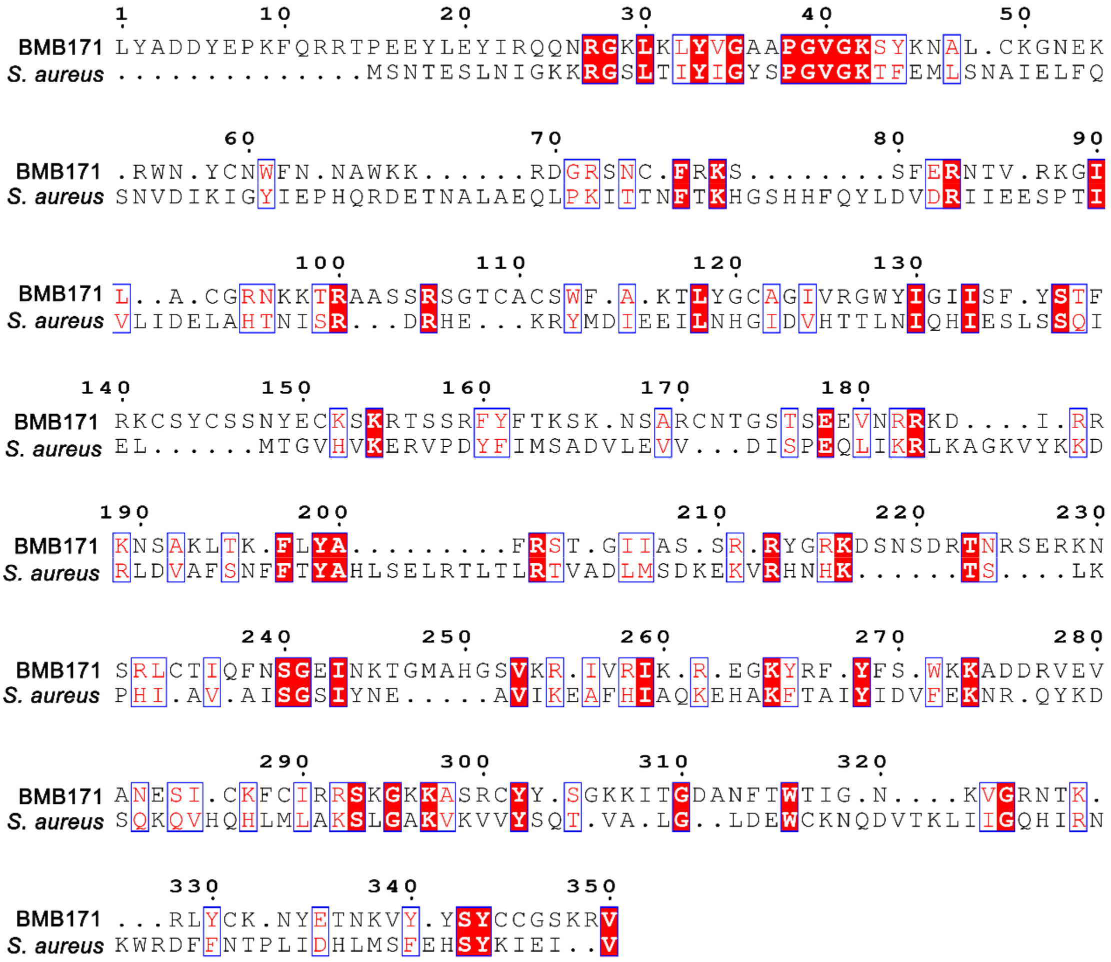

Supplementary Figure 1. Alignment of KdpD of BMB171 (*Bacillus thuringiensis* BMB171) with N-terminal domain of *S. aureus* (*Staphylococcus aureus* NCTC 8325). White letters shaded in red denote the conserved nucleotides. The similarity is only 9.1%.

Supplementary Figure 2

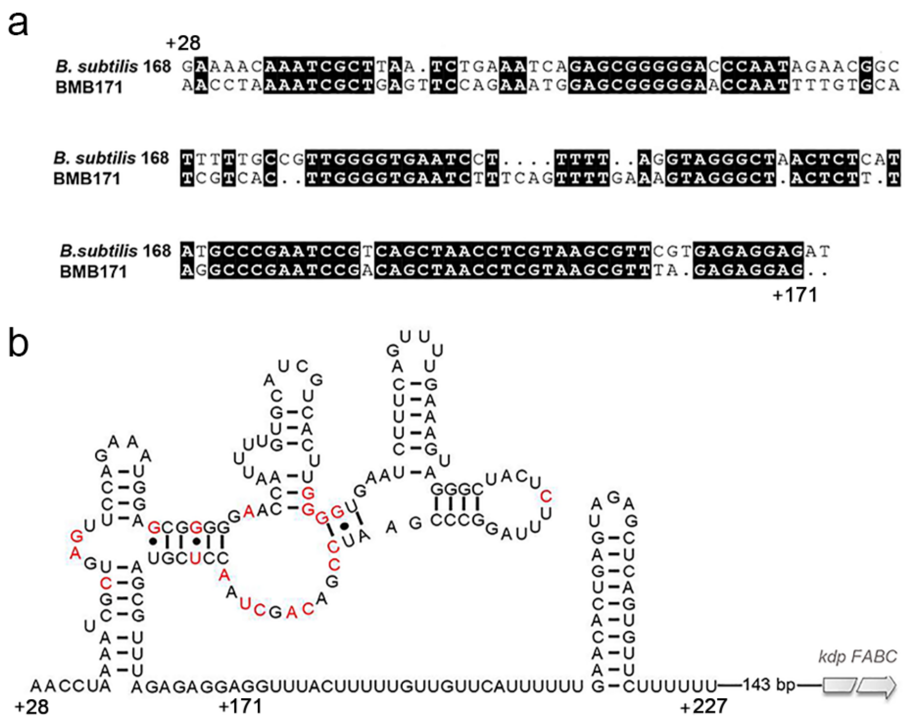

Supplementary Figure 2. A conserved c-di-AMP riboswitch encoding region within the 5'-UTR encoding region of *kdp* operon. a Sequence alignment of the aptamer domains in c-di-AMP riboswitch between BMB171 and *B. subtilis* 168, with white letters shaded in black denoting the conserved nucleotides. **b** The predicted secondary structure of c-di-AMP riboswitch. The distance from the 3' terminal of c-di-AMP riboswitch to the first nucleotide of the start codon is 143 bp.

Supplementary Figure 3

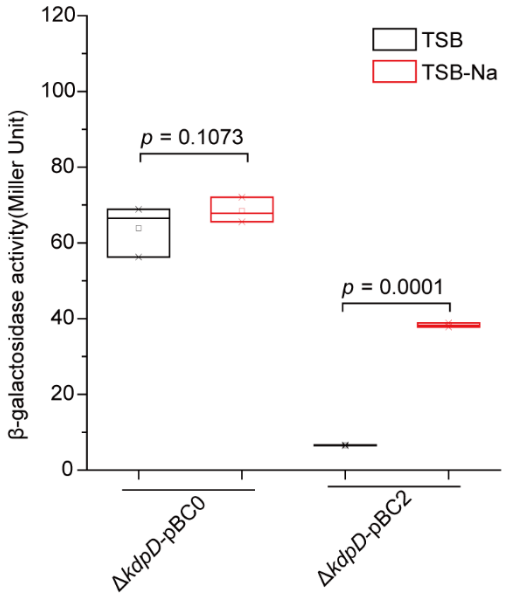

Supplementary Figure 3 The β-galactosidase activities for Δ*kdpD* carrying plasmids with (pBC2) and without the c-di-AMP riboswitch encoding region (pBC0) at different K^+ concentrations. Data are expressed as box-and-whisker plots, where the central lines denote medians, edges represent upper and lower quartiles and whiskers show minimum and maximum values. Data were subjected to one-way analysis of variance (ANOVA) using the Bonferroni test, $n = 3$, p-values are shown above each panel.

Supplementary Figure 4

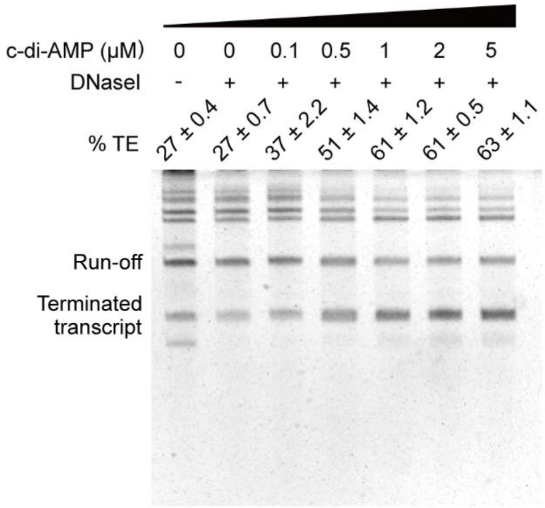

Supplementary Figure 4. ***In vitro*** **transcription assay conducted in the presence of various c-di-AMP concentrations ranging from 0 to 5 μM.**

Supplementary Figure 5

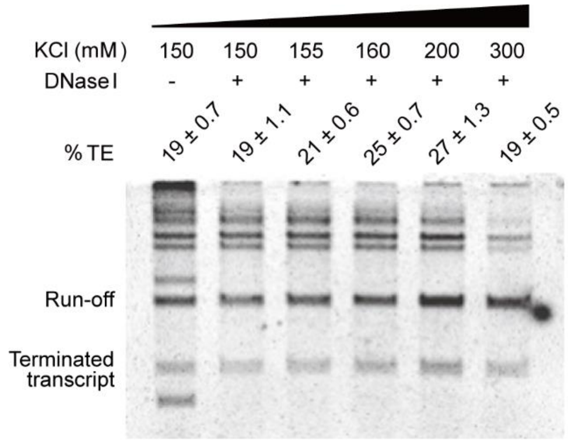

Supplementary Figure 5. ***In vitro*** **transcription assay conducted in the presence of various K^+ concentrations ranging from 150 to 300 mM.**

Supplementary Figure 6

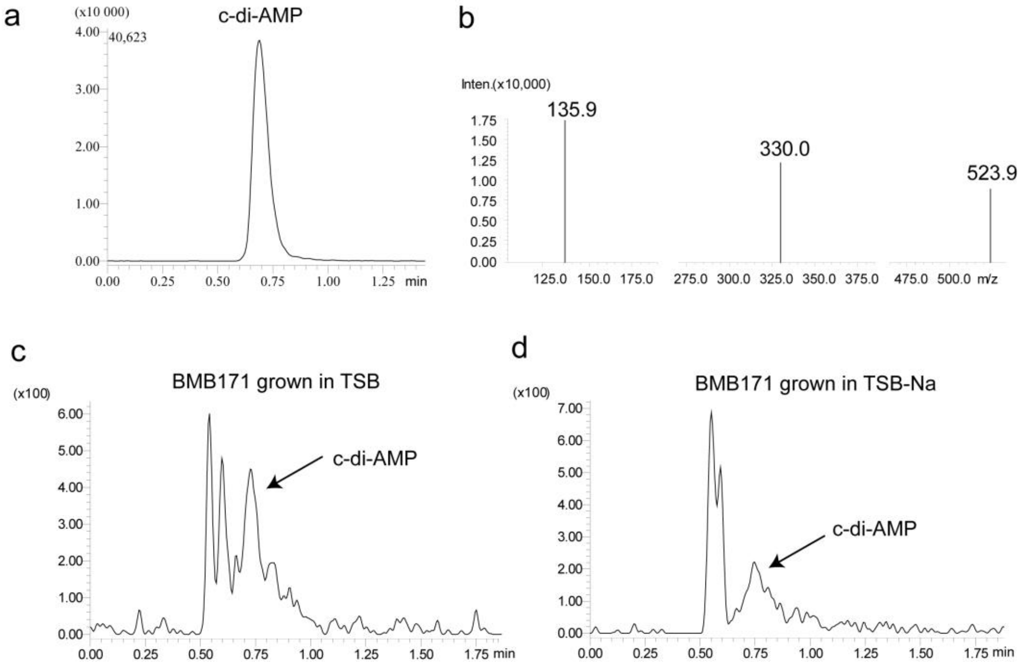

Supplementary Figure 6. Quantification of intracellular c-di-AMP concentration by LC-MS/MS. a LC-MS/MS chromatogram of the c-di-AMP standard detected using the *m/z* fragments at 135.9, 330.0 and 523.9. **b** Mass spectrum of daughter ions *m/z* 135.9, 330.0 and 523.9 from parent ions *m/z* 659.0 and 660.0. **c** and **d** Determination of c-di-AMP extracted from BMB171 strain grown in TSB and TSB-Na, respectively.

Supplementary Figure 7

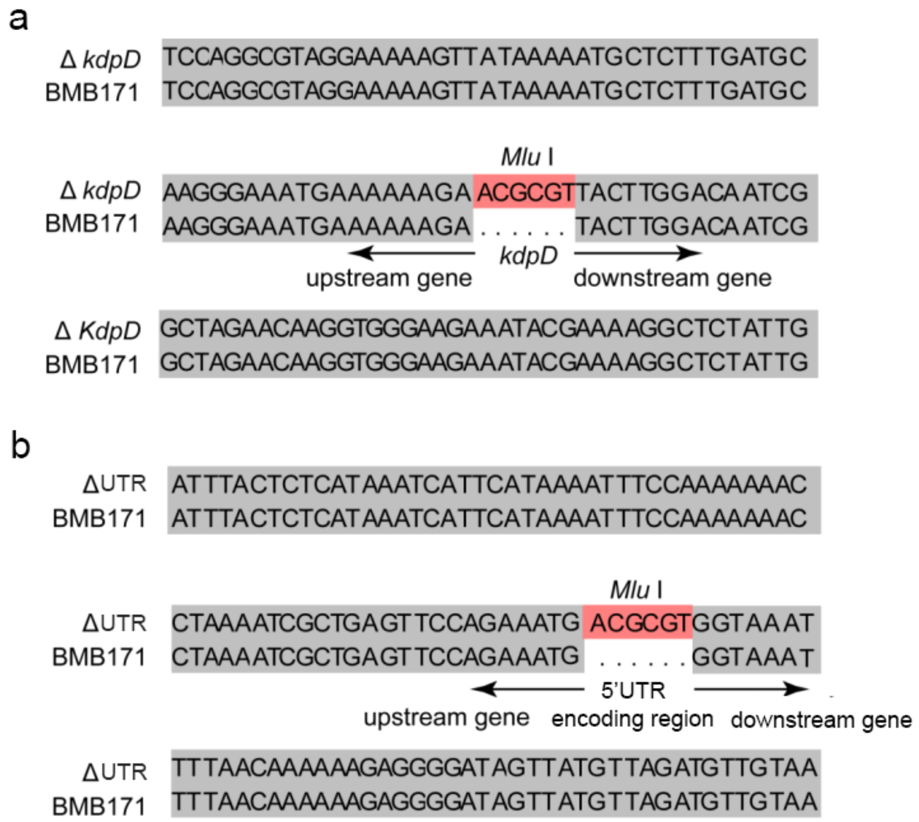

Supplementary Figure 7. Verification of Δ*kdpD* and ΔUTR by sequencing. **a** Sequence alignment of PCR products amplified from the Δ*kdpD* genomic DNA and the BMB171 genomic DNA using primer pair *kdpC*-F/ *kdpD*-DR (*Bam*H I). The PCR products (about upstream 100 bp and downstream 100 bp sequences of *kdpD*) were shown. **b** Sequence alignment of PCR products amplified from the ΔUTR genomic DNA and the BMB171 genomic DNA using primer pair UTR-UUF/UTR-DR (*Bam*H I). The PCR products (about upstream 100 bp and downstream 100 bp sequences of c-di-AMP riboswitch) were shown.

Supplementary Table 1. Primers used in this study

Primers	Consequence	Purposes	Origins
kdpA-F	CGTTAGATGGGGCGGTTACA	RT-qPCR	This work
kdpA-R	GTGACACGAAAAGGATGCGG		This work
kdpB-F	GGCGGTGATTTTTGTTCCGT		This work
kdpB-R	AAATCGGCAACGTCACAACG		This work
kdpC-F	AATCGGGCAAAACTTCACGG		This work
kdpC-R	ATATGCGATCCACCTGCACA		This work
gapdh-F	TTTTGCTAGCGCTTTCGCAG	RT-qPCR, internal control	This work
gapdh-R	TAGCGCCTGTTGTGAAGGTG		This work
16S-F	CCGCCCTTTAGTGCTGAAG		This work
16S-R	CTTGTGCGGGCCCCCGTCAATTC		This work
Primer-8	TTTTTTTTTTTTTTTT	5'-RACE	This work
UTR-R(5'-RACE)	AACTGAAAGATTCACCCCAAGTGACGA		
P*kdp*-F (*Nco* I)	CATGCCATGGAATAACAGTAATAATCTTCAATGTA	Plasmids pBC0 and pBC2 construction	This work
P*kdp*-R1 (*Bam*H I)	CGGGATCCGAGAGTAAATATACTCCTCTATAT	Plasmid pBC0 construction	This work
0647-R (*Bam*HI)	CGGGATCCTTTTTTGTTAAAATTTACCATTTAAATATTAAA	Plasmid pBC2 construction	This work
UTR-UF(*Hin*d III)	CCCAAGCTT GAGGATGAGGTACATGGAACTA	Plasmid pRP1028-UTR-UD construction	This work
UTR-UR (*Mlu* I)	CGACGCGT CATTTCTGGAACTCAGCGATTTTAG		This work
UTR-DF (*Mlu* I)	CGACGCGTGGTAAATTTTAACAAAAAAGAGGGGA		This work
UTR-DR(*Bam*H I)	CG GGATCC ATCCACCGCCGTTATTTCCAAG		This work
UTR-UUF	CTTCGTAAGCGTCTTGAGAGAGGAC	For c-di-AMP riboswitch encoding region deleted candidate screening	This work
pRP1028-R	GTGCGAATAAGGGACAGTGAAGAAGG		This work
kdpD-UF (*Sma* I)	TCCCCCGGG AATTAACATCACCACTGTCAGCG	Plasmid pRP1028-kdpD-UD construction	This work
kdpD-UR (*Mlu* I)	CG ACGCGT TCTTTTTTCATTTCCCTTGCATC		This work
kdpD-DF (*Mlu* I)	CGACGCGT TACTTGGACAATCGGCTAGAACA		This work
kdpD-DR(*Bam*H I)	CG GGATCC ATTATGCAGCTCGTATATGGACC		This work
kdpC-F	AATCGGGCAAAACTTCACGG	For chromosomal *kdpD* gene deleted candidate screening	This work
pRP1028-R	GTGCGAATAAGGGACAGTGAAGAAGG		
Eco-P	CAATGTAAGCGTTTAAATGAATG	*In vitro* transcription	This work
ZL-2R	TCCTTCTTTCACAACTTTATCTGACCAACT		This work

Supplementary Table 2. Strains used in this study

Strains	Relevant characteristics	Origins
E. coli DH5α	F- Φ80*lacZ*ΔM15 Δ(*lacZYA-argF*) U169 *rec*A1 *end*A1 *hsd*R17(rk^{-}, mk^{+}) *pho*A *sup*E44 *thi*-1 *gyr*A96 *rel*A1 λ-	Beijing TransGen Biotech Co., Ltd
BMB171	*B. thuringiensis* strain BMB171; an acrystalliferous mutant strain; high transformation frequency	3, 4
Δ*cdaA*	In frame deletion of *cdaA* in BMB171	5
Δ*disA*	In frame deletion of *disA* in BMB171	5
ΔUTR	In frame deletion of c-di-AMP riboswitch in BMB171	This work
Δ*kdpD*	In frame deletion of *kdpD* in BMB171	This work
BMB171-pBC0	Derivate strain of BMB171 carrying the pBC0 plasmid	This work
BMB171-pBC2	Derivate strain of BMB171 carrying the pBC2 plasmid	This work
Δ*cdaA*-pBC0	Derivate strain of Δ*cdaA* carrying the pBC0 plasmid	This work
Δ*cdaA*-pBC2	Derivate strain of Δ*cdaA* carrying the pBC2 plasmid	This work
Δ*disA*-pBC0	Derivate strain of Δ*disA* carrying the pBC0 plasmid	This work
Δ*disA*-pBC2	Derivate strain of Δ*disA* carrying the pBC2 plasmid	This work

Supplementary Table 3. Plasmids used in this study

Plasmids	Relevant characteristics	Purposes	Origins
pHT1K-*lacZ*	*B. thuringiensis-E. coli* shuttle plasmid; Amp^R Erm^R, pHT1K plasmid harboring the promoter-less *lacZ* gene, for β-galactosidase activity assay	β-Galactosidase assays	1
pBC0	*Kdp* promoter cloned between *Nco* I and *BamH* I sites of pHT1K-*lacZ,* for β-galactosidase activity assay	β-Galactosidase assays	This work
pBC2	*Kdp* promoter and c-di-AMP riboswitch coding region cloned between *Nco* I and *BamH* I sites of pHT1K-*lacZ*, for β-galactosidase activity assay	β-Galactosidase assays	This work
pRP1028	*B. thuringiensis-E. coli* shuttle plasmid; Amp^R Erm^R; containing *turbo-rfp* gene and an I-*Sce*I recognition site	Gene deletion	2
pSS4332	*B. thuringiensis-E. coli* shuttle plasmid; Km^R; containing *gfp* and I-*Sce*I restriction enzyme encoding gene	Gene deletion	2
pSS1827	The helper plasmid for conjugative transfer; Amp^R	Gene deletion	2
pRP1028-UTR-UD	pRP1028 with the upstream and downstream regions of c-di-AMP riboswitch, an intermediate plasmid in gene deletion experiments	c-di-AMP riboswitch encoding region deletion	This work
pRP1028-*kdpD*-UD	pRP1028 with the upstream and downstream regions of *kdpD* gene, an intermediate plasmid in gene deletion experiments	*kdpD* deletion	This work

Supplementary References

1. Wang, J. P., Ai, X. L., Mei, H., Fu, Y., Chen, B., Yu, Z.N. & He, J. High-throughput identification of promoters and screening of highly active promoter-5 '-UTR DNA region with different characteristics from *Bacillus thuringiensis*. *PLoS ONE*, **8,** e62960 (2013).
2. Janes, B. K. & Stibitz, S. Routine markerless gene replacement in *Bacillus anthracis*. *Infect Immun*, **74**, 1949-1953 (2006).
3. Li, L., Shao, Z., & Yu, Z. Transformation of *Bacillus thuringiensis* recipient BMB171 by electroporation. *Wei Sheng Wu Xue Tong Bao*, **27**, 331-334 (2000).
4. Li, L., Yang, C., Liu, Z., Li, F., & Yu, Z. Screening of acrystalliferous mutants from *Bacillus thuringiensis* and their transformation properties. *Wei Sheng Wu Xue Bao*, **40**, 85-90 (2000).
5. Zheng, C., Ma, Y., Wang, X., Xie, Y., Ali, M. K., & He, J. Functional analysis of the sporulation-specific diadenylate cyclase CdaS in *Bacillus thuringiensis*. *Front Microbiol*, **6**, 908 (2015).
